# Total serum IgE levels as predictor of the acquisition of tolerance in children with food allergy: Findings from a pilot study

**DOI:** 10.3389/fped.2022.1013807

**Published:** 2022-10-20

**Authors:** Giulia Dodi, Paola Di Filippo, Sabrina Di Pillo, Francesco Chiarelli, Marina Attanasi

**Affiliations:** Department of Pediatrics, Pediatric Allergy and Pulmonology Unit, University of Chieti-Pescara, Chieti, Italy

**Keywords:** food allergy, oral food challenge test, total IgE, egg allergy, tolerance acquisition

## Abstract

**Background:**

The gold standard to diagnose food allergy (FA) is a double-blind, placebo-controlled food challenge (OFC), even if it shows potential risk of severe allergic reactions for the patient and is time-consuming. Therefore, easier, and less invasive methods are needed to diagnose FA and predict the tolerance, changing the clinical practice.

**Aim:**

The main aim of this study was to assess whether the total IgE values at the diagnosis of FA were associated with the duration of the tolerance acquisition and thus of the food elimination diet.

**Methods:**

We retrospectively analyzed the medical records of 40 patients allergic to milk or egg who performed an OFC for the reintroduction of the causal food at the Pediatric Allergy and Respiratory Unit of the University of Chieti from January 2018 to December 2020.

**Results:**

We found a positive association of total serum IgE with the elimination diet duration (*β* = 0.152; CI, 95% 0.04–0.27) after adjusting for age, sex, and type of allergy (milk or egg). We also showed a significant correlation (*r* = 0.41 and *p*-value = 0.007) between the total IgE values and the duration of the elimination diet and a significant correlation between the casein specific IgE values at diagnosis of FA and the severity of the clinical presentation (*r* = 0.66; *p*-value 0.009).

**Conclusion:**

Total serum IgE at baseline, along with the downward trend of food-specific IgE levels (to milk or egg), may be useful in the prognostication of natural tolerance acquisition.

## Introduction

Cow’s milk is the most common and clinically important food allergen in children, likely because it is an important nutrient source in early childhood (1). However, the diagnosis of milk allergy is often difficult, with symptoms consistent with other conditions, including lactose intolerance and protein-induced enterocolitic syndrome. Nevertheless, growing up, many children acquire tolerance towards milk and they should be assessed for reintroduction (2). Egg allergy is the second most common food allergy (FA) in childhood, affecting 0.5%–8.9% of the pediatric population (3). Although the majority of children have outgrown their egg allergy by the age of 16, only 12%–50% reach the tolerance between the ages of 6 to 11 (4). Given the acquisition of tolerance over time in both egg and milk allergy, we decided to focalize our study on this topic and only consider patients affected by food allergy mediated by IgE-mediated mechanisms.

Oral food challenge (OFC) is considered the gold standard to diagnose FA and to assess the attained tolerance (5), but it is time-consuming, difficult to perform and related to the risk of severe allergic reactions. Therefore, easier, and less invasive laboratory methods have been evaluated to avoid the severe allergic reactions caused by OFC. The relationship between food-specific IgE levels and outcome of OFC was firstly investigated in a prospective study including 100 children with FA by Sampson et al. (6). The authors showed that the evaluation of food-specific IgE was useful for the diagnosis of symptomatic FA and for avoiding the need OFC in a considerable number of allergic children. For example, Komata et al. (7) found that specific IgE levels above 25.5 kUA/L for egg and 50.9 kUA/L for milk indicated a 95% risk of reaction to OFC. Specific IgE levels ≤2 kUA/L to milk, egg, or peanut (or ≤5 kUA/L to peanut without history of previous reaction) 41 were associated with 50% likelihood of negative OFC. Therefore, children with these specific IgE cut-offs were appropriate candidates for OFC to investigate the acquisition of tolerance (8). Further prospective studies developed significant FA probability curves to predict OFC test failure (9, 10). Mehl et al. (11) retrospectively analyzed 992 OFCs performed in 501 children with median age of 13 months, showing a significant correlation between the specific IgE/total IgE ratio and the outcome of OFCs for milk, egg, and wheat, although without benefit compared with the specific IgE alone. However, the correlations between specific IgE and total IgE levels are not linear, given that total IgE level usually follows a lognormal distribution while the specific IgE level does not, therefore the calculation of the ratio might be too simple to accurately reflect the true relationship between the two factors. Horimukai et al. (5) examined retrospectively the medical records of 337 and 266 patients who underwent OFCs for 3.5 g boiled hen's egg white and 3.1 ml raw cow's milk, respectively. The authors found that children with higher total IgE levels were significantly less responsive during OFC; indeed, they explained that nonspecific IgE could suppress specific IgE-mediated activation of basophils *in vitro* (12), affecting the clinical severity of patients with food, drug and Hymenoptera venom allergies (13, 14). The previous findings identified clinical and laboratory factors that could predict the negativity of the OFCs reducing the number of unnecessary tests. Differently, in our study we decided to evaluate whether total IgE levels could be considered as a marker of the acquisition of tolerance after an elimination period of the causal allergen in children with milk or egg allergy.

The main aim of this pilot study was to assess the association between total IgE levels at the diagnosis of FA and the duration of the causal food elimination diet in order to establish the best timing to perform an OFC without severe allergic reactions.

## Methods

We carried out a retrospective study to investigate the clinical and laboratory parameters in milk and egg allergic children. The inclusion criteria were:

• children diagnosed with IgE-mediated allergy to milk (group 1) or egg (group 2) who were on a specific food elimination diet; the diagnosis was based on clinical history and positive food-specific IgE values;
• OFC test performed to assess for tolerance acquisition, in the Pediatric Allergy and Respiratory Unit of the University of Chieti from January 2018 to December 2020;
• available total IgE values at the diagnosis (time 0), specific IgE for egg and milk and recombinant milk allergens (lactalbumin, lactoglobulin and casein recombinant allergens) at the diagnosis (time 0) and at the time of OFC (time 1).

An accurate family and personal medical history was collected by the parental interview and by consulting medical records. Symptoms of allergy at the onset and children age at the onset of FA, at the beginning of food elimination diet and of food reintroduction were obtained. To better describe our study population, symptoms at the allergy onset were classified as localized (cutaneous, gastrointestinal, upper respiratory) or systemic (anaphylaxis) and characterized according to the severity of symptoms (15). The definition of anaphylaxis is the one of WHO 2020 (16).

(1) Mild (grade 1): isolated local reactions of the skin or mucous membranes in the site of contact with the allergen.
(2) Moderate (grade 2): allergic reactions involving skin areas away from the site of contact with the allergen, the upper airways, and/or the gastrointestinal tract.
(3) Severe (grade 3): severe allergic reactions, potentially at risk of life, which include signs which include cardiovascular, neurological, bronchial and/or laryngeal signs and symptoms.

All the patients involved in the study were selected to perform OFC when their food-specific IgE results fell below 15 KUA/L that we usually use as a cut-off; furthermore, none of them presented recent clinical reactions to accidentally administered causal food in the previous twelve months. In our center, we usually visit allergic patients annually and perform food-specific IgE dosages each time (17).

Primary outcome was to investigate the association between total IgE levels at baseline and the duration of elimination diet (years).

Secondary outcomes were:

- to assess the difference between food-specific IgE levels (milk or egg) at the diagnosis of FA and at the time of food re-introduction;
- to assess the association of different recombinant milk allergens with the severity of the clinical manifestations at the diagnosis.

Written informed consent was obtained from parents or legal representatives of all children. The study was approved by the local Ethical Committee of the University of Chieti, and it was conducted in compliance with ethical principles based on the Declaration of Helsinki.

### Statistical analysis

Continuous data were presented as mean and standard deviation (SD) or median and range 5%–95%. Categorical data were presented as absolute numbers and percentages. Shapiro-Wilk test was used to investigate the normality of the distribution of all variables. Given the non-normal distribution of total IgE, the logarithmic transformation has been applied to normalize this variable. Nonparametric tests, such as Wilcoxon test or Mann-Whitney test, were used for continuous variables, while the *χ*^2^ test for categorical variables. Pearson's correlation was used to assess the correlation between total IgE levels and duration of elimination diet.

In addition, a multivariate linear regression model was performed to investigate the association between total IgE and duration of elimination diet after adjusting for potential confounding factors, such as sex, age at the diagnosis and type of allergy. Spearman's correlation was performed to assess the correlation of different recombinant milk allergens (lactalbumin, lactoglobulin and casein IgE values) with the severity of symptoms at the diagnosis. *P-*value <0.05 was considered for the significance of our statistical reports.

Statistical analysis was conducted using Stata 15 Software for Statistics and Data Science for Windows

## Results

We enrolled 40 patients: 14 (35.0%) were on milk elimination diet and underwent milk OFC, while 26 (65.0%) were on egg elimination diet and performed egg OFC. Clinical and laboratory characteristics of our population were reported in Table 1.

**Table 1:** Clinical and laboratory characteristics of our population.

	Patients with milk allergy	Patients with egg allergy	Total population	*p*–value
Sex (%)				
Male	10 (71.43)	17 (65.38)	27 (67.50)	0.697*
Female	4 (28.57)	9 (34.62)	13 (32.50)	
Age at baseline (years)	0.77 (0.25–1.6)	1 (0.3–4.0)	0.9 (0.25–4)	0.585**
Total IgE at baseline (KU_A_/L)	102 (2–4,888)	179.5 (11.9–777)	179.5 (2–4,888)	0.460***
Diet duration (years)	4.28 (1–13)	5.67 (1.3–13.1)	5.6 (1–13.1)	0.497**
OFC, positive (%)	1 (7.14)	3 (11.54)	4 (10)	0.658*
Specific serum IgE at FA (KU_A_/L)				
Milk	8.9 (0.0–83.4)	–	–	–
Egg yolk	–	1.06 (0–10.9)	–	–
Egg white	–	4.0 (0.25–45.13)	–	–
Casein	2.8 (0.0–31.7)	–	–	–
Alfa-lactalbumin	1.94 (0–83.4)	–	–	–
Beta-lactoglobulin	2.3 (0–74.4)	–	–	–
Specific serum IgE at OFC (KUA/L)				
Egg yolk	–	0.23 (0.0–2.94)	–	–
Egg white	–	0.71 (0.01–6.22)	–	–
Casein	0.16 (0.0–10)	–	–	–
Alfa-lactalbumin	0.05 (0–15)	–	–	–
Beta-lactoglobulin	0.10 (0–7.3)	–	–	–
Delta change specific IgE at FA and OFC				
Egg yolk	–	0.46 (−2.26–7.96)	–	–
Egg white	–	3.09 (−1.58–44)	–	–
Casein	0.83 (−0.19–31.05)	–	–	–
Alfa-lactalbumin	1.84 (−1.9–68.4)	–	–	–
Beta-lactoglobulin	2.20 (0–74.2)	–	–	–
Symptoms type and severity (%)				
* *Cutaneous	7 (50.00)	12 (46.2)	19 (47.50)	0.641*
* *Gastro-intestinal	7 (50.00)	8 (30.7)	15 (37.50)	<0.05*
* *Upper Airways	1 (7.14)	3 (11.54)	4 (10.00)	0.950*
Anaphylaxis	2 (14.29)	3 (11.54)	5 (12.50)	0.800*
Mild	2 (14.29)	10 (38.46)	12 (30.00)	0.112*
Moderate	8 (57.14)	12 (46.15)	20 (47.50)	0.370*
Severe	4 (28.57)	4 (15.38)	8 (20.00)	0.320*

Data are presented as absolute numbers and percentages (%) or median and ranges.

FA, food allergens; OFC, oral food challenge. Bold formatting to values where the *p*-value is <0.05.

* *p*-values from Pearson’s Chi-square (*χ*^2^) test.

** *p*-values from Unpaired t-Test.

*** *p*-values from Mann Whitney *U* test.

Clinical manifestations at the onset occurred at the median age of 9 months in the group 1. Symptoms described by parents were skin reactions in 7/14 (50.0%) patients, gastrointestinal disorders in 7/14 (50.0%) patients, upper respiratory involvement in 1/14 (7.1%) patients, anaphylaxis in 2/14 (14.3%) patients. Symptoms were mild in 2/14 (14.3%), moderate in 8/14 (57.1%) and severe in 4/14 (28.6%) children.

Clinical manifestations at the onset occurred at the median age of 12 months in the group 2. Symptoms reported by parents were skin reactions in 12/26 (46.2%) patients, gastrointestinal disorders in 8/26 (30.8%) patients, bronchial involvement 3/26 (11.5%) patients, anaphylaxis in 3/26 (11.5%) patients. Symptoms were mild in 10/26 (38.5%), moderate in 12/26 (46.15%) and severe in 4/26 (15.4%) children.

One of 14 (7.1%) milk OFC resulted positive; therefore, 13 children in the group 1 (92.9%) reached the tolerance and started a free diet. Three of 26 (11.5%) egg OFCs were positive; therefore, 23 children in the group 2 (88.5%) reached the tolerance and started a free diet.

The median duration of food allergy (from onset of symptoms to reintroduction of food) was 4.3 years in patients with milk allergy and 5.7 years in patients with egg allergy. We obtained a positive association between total IgE levels and duration of the elimination diet, after adjusting for age, sex, and type of allergy as well (*β* = 0.153; CI, 95% 0.04–0.27).

**Figure 1:**
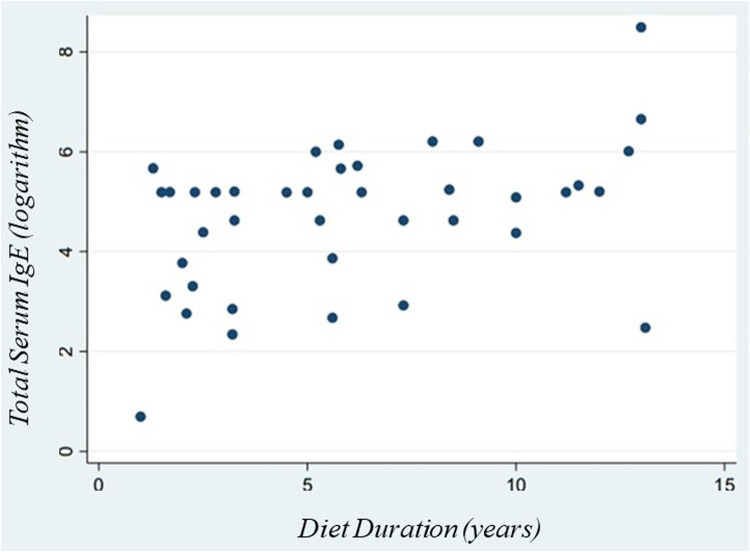
Correlation between total serum IgE and diet duration.

Additionally, we confirmed the linear correlation between total IgE levels at the diagnosis (expressed in logarithmic scale) and the duration of the elimination diet in the study population by using Pearson's correlation (*r* = 0.41; *p*-value = 0.007) (Figure 1).

As regards the secondary outcomes, we also found a significant difference among specific IgE values for casein, α-lactalbumin and β- lactoglobulin at the diagnosis and those obtained at the time of OFC (*p* = 0.008; *p* = 0.02; *p* = 0.03; respectively). We also found a significant difference among specific IgE values for egg white and yolk at the diagnosis and those obtained at the time of OFC (*p* < 0.001; *p* < 0.001; respectively). Supplementary Tables S1 and S2 reported these data.

In addition, we observed a significant correlation between specific IgE values for casein at the diagnosis and the severity of symptoms at the onset of FA (Rho = 0.66; *p*-value 0.009). On the contrary, no correlation of specific IgE for α-lactalbumin and β-lactoglobulin with severity of symptoms at the onset of allergy was found.

## Discussion

In the literature, cow's milk allergy appears during early infancy since the first two months of life (18, 19). Egg allergy generally appears with the first intake of food in the second half of the first year of life, at about 1 year of age (20). In our study population, the median age of onset was 9 months for cow's milk allergy and 12 months for egg allergy, comparable to literature findings.

In group 1, 50.0% of milk allergic patients presented cutaneous manifestation, whereas the percentage of cutaneous manifestation reported in the literature is higher. For example, Martorell et al. (21) found the most frequent clinical signs were cutaneous in 94% of milk allergy cases. In agreement with the literature (22), in group 2 the symptoms at the onset were more commonly cutaneous (42.3% of children showed erythema, itching, hives and angioedema), while the gastrointestinal disorders such as vomiting, diarrhea and abdominal pain were less frequent (30.8% of cases), as well as upper respiratory (7.7% of cases) and systemic involvement (anaphylaxis in 11.5% of children).

The median duration of milk allergy in our sample was 4.3 years. This finding differs slightly from the data in the literature, which reported an allergy resolution rate of 45%–50% within the first year of life, 60%–75% within 2 years, and 85%–90% within the third year of life (23). The median duration of egg allergy in our sample was 5.7 years. In children, the prognosis of egg allergy is generally good (20). In previous studies Boyano et al. (24) reported a clinical resolution in 50% of cases within 3 years of age, and in 66% of cases within five years (25). More recently, Clark et al. (25) found that nearly 1/3 of their patients had resolved allergy to well-cooked egg at 3 years and 2/3 at 6 years. Having discussed the results concerning the characteristics of the population, we now focus on the objectives of our study. In this study, in fact, we assessed total IgE at the time of diagnosis as a predictor of the time required for acquiring the tolerance for the causal food allergen.

To date, OFC remains the gold standard to diagnose FA as indicated by EAACI food allergy and anaphylaxis guidelines (26). However, in clinical practice easier and less invasive laboratory methods would be useful in the follow up of allergic patients. Sampson (6) and Komata (7) separately confirmed that quantification of food-specific IgE could avoid probably positive OFC tests thus reducing adverse reactions. At the same time, children with specific IgE ≤ 2 kUA/L to milk, egg, or peanut (or ≤5 kUA/L to peanut without history of previous reaction) were appropriate candidates for OFC to investigate the resolution of FA, because these specific IgE cut-offs were associated with 50% likelihood of negative OFC (7). As in the literature there are several reports about specific IgE role in assessment of acquisition of tolerance (6, 7), we hypothesized we could find a relationship also between total IgE values and acquired tolerance. With this aim, we analyzed the medical records of milk and egg allergic patients who underwent OFC in our center and found an association between total IgE values and diet duration.

Currently, the evaluation of total IgE is only considered a supplemental diagnostic measure for allergic diseases, because of the relatively low sensitivity and specificity and the undefined cost-effectiveness (15). In the literature, we found little evidence to support our result.

Conversely, Beigelman et al. (8) found that higher total IgE levels were associated with an increased likelihood of negative oral milk challenge test, but the authors did not provide an explanation. Al Mughales et al. (27) stated that total IgE evaluation was not efficient as a diagnostic test, especially in food allergies. More recently, an observational retrospective study included 253 children with median age of 8 years affected by milk or egg allergy who underwent OFCs. The authors concluded that total IgE measurement was not recommended because the ratio specific/total IgE was not a better predictor of the outcome of OFCs than food-specific IgEs alone (28). Furthermore, we know that total IgE levels naturally tend to increase over time (29), often coinciding with the acquisition of tolerance. Regarding the previous data and the seeming contradiction with our results, we noted that we performed total IgE dosage at the diagnosis of FA while in the previous studies it was performed at the moment of OFC and also that our sample size is smaller than that of the other studies and this fact could affected the obtained results.

We also found a substantial reduction of specific IgE values from diagnosis to the execution of OFC. It is well known that the greater the serum IgEs level, the greater the probability of allergic reactions (6, 7). In 2018 in their systematic review Calvani et al. (30) suggested that raw egg allergy seems very likely if food-specific IgEis ≥ 1.7 kUA/L in children aged <2 years, while in children 2 years or older the suggested cut-off is ≥7.3 kUA/L (30). For example, Dang et al. found that an egg white food-specific IgE cut-off of 11.0 kUA/L had a specificity of 95% and a sensitivity of 45%, to predict 50% of probability of acquired tolerance (31). Therefore, specific IgE are commonly used in the follow161 up of a child's elimination diet, and their values associate with the likelihood of severe allergic reactions during OFC (32, 33). However, a standardized threshold value of food-specific IgEs to predict the outcome of OFC and the acquisition of tolerance was not definitely established yet. Additionally, the cut-off points predictive of clinical reactivity change over time and depend on the moment of follow-up (34). Briefly, with our results we support the fact that the decrease of specific IgE values in the allergic patient's follow-up could guide the physician in the timing of the food reintroduction, as well as described in the literature.

As a last point, we found a significant correlation between specific IgE for casein and the severity of the symptoms at the onset of FA. Casein is the main protein allergen in cow's milk (34) and several studies have recognized specific IgE for casein as the best predictive factor of positive OFC (34–37). Martorell et al. (34) in a prospective 4-year follow-up study including 170 cow’s milk allergic patients, described that specific IgE levels to casein 0.97 kUA/L at 12 months, 1.22 kUA/L at 18 months, 3 kUA/L at 24 months, 2.39 kUA/L at 36 months, 2.73 kUA/L at 48 months predicted the clinical reactivity, respectively.

This is the first study which examined the association between total serum IgE at baseline, and the duration of elimination diet in milk and egg allergic children. However, some methodological limitations should be discussed. Firstly, a retrospective study design could lead to bias related to the type of study. However, we considered several measures to minimize bias and to increase the validity of our findings. To minimize potential selection bias, we fixed clearly defined inclusion/exclusion criteria identifying the population of interest. To minimize potential measurement bias, data were collected by a trained research pediatrician using a predefined questionnaire. Secondly, the small sample size could have affected the power of the study. Lastly, although we adjusted for several confounders, we might not have had information on all possible confounding factors. Therefore, our findings should be confirmed in observational prospective studies with a large sample of allergic children rightly selected who underwent double-blind placebo controlled OFCs.

In conclusion, we would suggest that total IgE values could be useful in managing children with FA. Particularly, this simple and cheap test should be considered to guide the decision of the duration of the elimination diet and the timing for food reintroduction. We also confirmed the utility of specific IgE for casein to predict the severity of the clinical manifestations. In clinical practice, the allergy tests commonly used to support food allergy diagnosis are skin prick test and specific IgE levels, with total IgE levels, which can also be used to monitor the spontaneous resolution of food allergy over time.

## Data Availability

The raw data supporting the conclusions of this article will be made available by the authors, without undue reservation.
