# Supplementary material for: Total serum IgE levels as predictor of the acquisition of tolerance in children with food allergy: Findings from a pilot study
Source: Front Pediatr. 2022 Oct 20;10:1013807. doi: 10.3389/fped.2022.1013807 (PMC9630731; doi:10.3389/fped.2022.1013807)
Supplement: Supplementary file 1 [file Table1.docx]

Supplementary table 1 and supplementary table 2 providing specific IgE values and gravity of symptoms of all patients.

Table 1. IgE values and onset symptoms in egg allergic patients.

|  | **Yolk IgE at diagnosis** | **Yolk IgE at OFC** | **Egg white IgE at diagnosis** | **Egg white IgE at OFC** | **Mild**  **Symptoms** | **Moderate**  **Symptoms** | **Severe**  **Symptoms** |
| --- | --- | --- | --- | --- | --- | --- | --- |
| 1. | 1.16 | 0.35 | 5.86 | 1.23 | 0 | 1 | 0 |
| 2. | 1.06 | 0.09 | 2.45 | 0.31 | 1 | 0 | 0 |
| 3. | 1.41 | 0.225 | 4.05 | 0.71 | 1 | 0 | 0 |
| 4. | 0.17 | 0.1 | 0.48 | 0.1 | 1 | 0 | 0 |
| 5. | 0.39 | 2.65 | 1.36 | 2.94 | 0 | 1 | 0 |
| 6. | 0.6 | 0.11 | 2.7 | 0.33 | 0 | 1 | 0 |
| 7. | 0.12 | 0.01 | 0.4 | 0.01 | 0 | 0 | 1 |
| 8. | 10.9 | 2.94 | 30.4 | 6.22 | 0 | 1 | 0 |
| 9. | 6.24 | 0.45 | 44.2 | 1.23 | 1 | 0 | 0 |
| 10. | 2.94 | 0.42 | 27.8 | 0.79 | 0 | 1 | 0 |
| 11. | 3.76 | 1.44 | 24.9 | 0.63 | 0 | 1 | 0 |
| 12. | 1.06 | 0.3 | 4.005 | 0.8 | 0 | 0 | 1 |
| 13. | 1.32 | 0.15 | 2.88 | 0.23 | 1 | 0 | 0 |
| 14. | 3.05 | 0.11 | 3.06 | 0.16 | 1 | 0 | 0 |
| 15. | 1.06 | 0.74 | 4.52 | 1.42 | 1 | 0 | 0 |
| 16. | 2.35 | 0.81 | 3.98 | 0.91 | 0 | 1 | 0 |
| 17. | 0.07 | 0.05 | 0.25 | 0.22 | 0 | 1 | 0 |
| 18. | 1.15 | 0.83 | 6.89 | 1.7 | 0 | 1 | 0 |
| 19 | 3.59 | 0.1 | 28.7 | 0.43 | 0 | 1 | 0 |
| 20. | 0.08 | 0.225 | 0.58 | 0.71 | 0 | 0 | 1 |
| 21. | 0.12 | 0 | 0.97 | 0.04 | 1 | 0 | 0 |
| 22. | 0.99 | 0.78 | 45.13 | 1.13 | 1 | 0 | 0 |
| 23. | 1.06 | 1.42 | 4.005 | 2.77 | 0 | 1 | 0 |
| 24. | 0.35 | 0.15 | 6.94 | 0.88 | 0 | 1 | 0 |
| 25. | 0.48 | 0.08 | 4.03 | 0.4 | 0 | 0 | 1 |
| 26. | 0.17 | 0.08 | 0.67 | 0.28 | 1 | 0 | 0 |

Table 2. IgE values and onset symptoms in milk allergic patients.

|  | **Casein at diagnosis** | **Casein at OFC** | **α-lactalbumin at diagnosis** | **α-lactalbumin at OFC** | **β-lactoglobulin at diagnosis** | **β-lactoglobulin at OFC** | **Mild**  **Symptoms** | **Moderate**  **Symptoms** | **Severe**  **Symptoms** |
| --- | --- | --- | --- | --- | --- | --- | --- | --- | --- |
| 1. | 12 | 0.19 | 27.7 | 0.97 | 1.23 | 0.11 | 0 | 1 | 0 |
| 2. | 3 | 0.13 | 50.3 | 0.03 | 74.4 | 0.2 | 0 | 0 | 1 |
| 3. | 0.08 | 0 | 1.8 | 0.16 | 0.04 | 0 | 0 | 1 | 0 |
| 4. | 2.86 | 0.07 | 2.07 | 0 | 2.88 | 0.07 | 1 | 0 | 0 |
| 5. | 0.01 | 0.19 | 0.7 | 2.58 | 0 | 0.01 | 0 | 1 | 0 |
| 6. | 0.3 | 0.05 | 0.07 | 0.02 | 2.25 | 0.07 | 0 | 1 | 0 |
| 7. | 4.28 | 2.9 | 10.1 | 2.04 | 3.77 | 3.34 | 0 | 1 | 0 |
| 8. | 0 | 0 | 0 | 0 | 0 | 0 | 0 | 1 | 0 |
| 9. | 0.01 | 0 | 0.14 | 0 | 0.22 | 0 | 0 | 1 | 0 |
| 10. | 31.7 | 0.65 | 0.04 | 0.01 | 2.01 | 0.04 | 0 | 0 | 1 |
| 11. | 2.73 | 0.37 | 0.63 | 0.03 | 2.36 | 0.25 | 0 | 1 | 0 |
| 12. | 26 | 0.29 | 4.04 | 0.36 | 7.01 | 0.34 | 0 | 0 | 1 |
| 13. | 0.31 | 0.12 | 2.35 | 0.1 | 2.31 | 0.1 | 1 | 0 | 0 |
| 14. | 10 | 10 | 83.4 | 15 | 37.8 | 7.3 | 0 | 0 | 1 |
